# Coexistence of diploid and triploid hybrid water frogs: population differences persist in the apparent absence of differential survival

**DOI:** 10.1186/1472-6785-10-14

**Published:** 2010-05-27

**Authors:** Ditte G Christiansen, Christian Jakob, Martina Arioli, Sandra Roethlisberger, Heinz-Ulrich Reyer

**Affiliations:** 1 Institute of Evolutionary Biology and Environmental Studies, University of Zurich, Winterthurerstrasse 190, CH-8057 Zurich, Switzerland

## Abstract

**Background:**

The role of differential selection in determining the geographic distribution of genotypes in hybrid systems has long been discussed, but not settled. The present study aims to asses the importance of selection in structuring all-hybrid *Pelophylax esculentus *populations. These populations, in which the parental species (*P. lessonae *with genotype LL and *P. ridibundus *with genotype RR) are absent, have pond-specific proportions of diploid (LR) and triploid (LLR and LRR) genotypes.

**Results:**

With data from 12 Swedish ponds, we first show that in spite of significant changes in genotype proportions over time, the most extreme ponds retained their differences over a six year study period. The uneven distribution of genotypes among ponds could be a consequence of differential selection varying among ponds (selection hypothesis), or, alternatively, of different gamete production patterns among ponds (gamete pattern hypothesis). The selection hypothesis was tested in adults by a six year mark-recapture study in all 12 ponds. As the relative survival and proportion of LLR, LR and LRR did not correlate within ponds, this study provided no evidence for the selection hypothesis in adults. Then, both hypotheses were tested simultaneously in juvenile stages (eggs, tadpoles, metamorphs and one year old froglets) in three of the ponds. A gradual approach to adult genotype proportions through successive stages would support the selection hypotheses, whereas the presence of adult genotype proportions already at the egg stage would support the gamete pattern hypothesis. The result was a weak preference for the gamete pattern hypothesis.

**Conclusions:**

These results thus suggest that selection is of little importance for shaping genotype distributions of all-hybrid populations of *P. esculentus*, but further studies are needed for confirmation. Moreover, the study provided valuable data on genotype-specific body lengths, adult survival and sex ratios.

## Background

Species coexistence is believed to be niche-based [1]. However, for hybrid complexes, opinions differ as to whether environment-specific differential selection is important for the geographic distribution and diversity of hybrid genotypes. Theory has developed along two lines, both represented by two opposing, but not mutually exclusive models. Within each line, the first model assumes environment-specific differential selection on hybrids, whereas the alternative model assumes that selection on hybrids does not vary among environments. The first line applies to the mixture of hybrids and their parental species (the tension zone model [2] and the bounded hybrid superiority model, e.g. [3]). The second line concerns hybrid clones (the frozen niche variation model [4] and the general purpose genotype model, e.g. [5]). However, for hybrids that are neither sympatric with their parental species, nor clonal, theories have not been formulated and, consequently, the role of differential selection in determining the geographic distribution and diversity of such hybrids is unknown.

The edible frog, *Pelophylax esculentus *(called *Rana esculenta *until Frost et al. [6]) constitutes an example of a hybrid that can form all-hybrid populations that are neither sympatric with parental species [7,8] nor clonal [9]. These hybrids demonstrate such extreme hybrid superiority that parental species genotypes continuously arising from hybrid × hybrid matings are constantly outcompeted [8,10] and thus virtually absent among adults. Still, various genotype classes are present, as the hybrids include both diploid and triploid forms. Genotype proportions have been observed to vary among ponds, and it remains to be assessed whether differential selection among ponds is responsible.

Within the genus of water frogs, *Pelophylax*, the edible frog, *P. esculentus *(genotypes LLR, LR and LRR), arose and still arises by matings between the pool frog, *P. lessonae *(Camerano, genotype LL), and the marsh frog, *P. ridibundus *(genotype RR, i.e. [11]). As indicated by the names, the two parental species have different habitat preferences within their largely overlapping distribution areas that cover most of Europe. The smaller *P. lessonae *lives in pools and ponds while the larger *P. ridibundus *prefers lakes and river-influenced water bodies [12,13]. The *P. esculentus *hybrids have the broader habitat tolerance and usually co-occur with at least one of the parental species.

**Figure 1:**
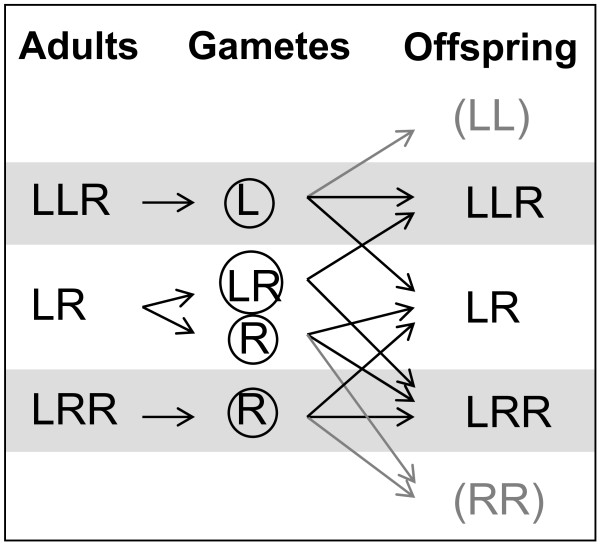
**Simplified illustration of gamete production and population maintenance of all-hybrid *P. esculentus *populations**. Note that the frogs mostly produce offspring with genotypes different from their own. LL and RR die before sexual maturity; LRR males (and LLRR frogs) are formed rarely, because LR sperm (as opposed to LR eggs) is rare.

The hybrids reproduce by hybridogenesis, which implies that genetic recombination does normally not take place between L and R genomes in hybrids. Instead, gametes contain one or the other genome, or both, but not a mixture. Hybrids are thus formed anew every generation by the fusion of two gametes with different genomic contents. In the all-hybrid populations of Southern Sweden that were investigated in this study, LLR frogs of both sexes make mostly L gametes (LLR females also make low proportions of LL eggs), LRR of both sexes make R gametes, LR females make LR and some R eggs while LR males make R and rarely also LR or L sperm (Figure 1, [7,8,14]). When two L or two R gametes combine, offspring with parental species genotypes (LL and RR) arise, but under natural conditions they die before sexual maturity [8,10]. Sex determination is an XX-XY system with a male-determining Y factor located in one L genome in males [14]. As a consequence, LRR males are rare, except in ponds with high frequencies of LR sperm [14]. Tetraploids are also rare [7]. The remaining five hybrid genotypes, LLR and LR males, LLR, LR and LRR females, are frequent in almost all ponds [7].

Because the various genotypes propagate each other rather than themselves (cf. Figure 1), the populations are self-sustaining and should constantly be drawn to a gamete pattern-determined stable equilibrium [14,15]. Yet, variation in the proportions of LLR, LR and LRR among ponds has been observed [7,10]. It remains to be established how such variation can be generated; especially if it does not result from stochastic events, but is maintained over time. Two hypotheses have been proposed to explain such persistent equilibrium differences: 1) Variation in selection regimes among ponds [7]; here called the selection hypothesis. 2) Variation in gamete patterns among ponds [14]; here called the gamete pattern hypothesis. A third possibility is that both selection and gamete pattern contribute to the variation in genotype compositions among ponds. They could either act antagonistically, or pond-specific gamete patterns could be adapted to the local selection regime.

The selection hypothesis is based on the observation of differences among *P. lessonae*, *P. ridibundus *and *P. esculentus *in adult habitat preference and in larval performance under various ecological conditions, as found by a variety of studies [13,16,17]. Ecological differences could thus also exist within hybrid genotypes, i.e. among LLR, LR and LRR. Such differences could either be a consequence of a dosage effect, as observed in morphometry (callus size divided by tibia length), where there is a cline from LL through LLR, LR and LRR to RR [7,18-20], or as a consequence of triploids having larger cells, as observed in erythrocytes [7,21]. The only study comparing fitness of LLR, LR and LRR in different habitats was, however, not very conclusive: The prevalence of adult LLR was positively correlated with human constructions and that of LR adults with forest around the breeding pond, but the majority of ecological parameters measured were not significantly correlated with genotype proportions [7].

The gamete pattern hypothesis is based on a study showing a tendency for variation in gamete patterns among ponds [14]. The sample size was not sufficiently large to document significant differences, given the large variation among individuals, but the trend pointed in a direction that could explain the difference in genotype proportions between so-called "normal" and "LRR-rich" populations.

Distinguishing between the selection and gamete pattern hypotheses does not only help us understand how these intriguing all-hybrid populations function; it has consequences for our perception of this and other breeding systems. The selection hypothesis would suggest that the all-hybrid populations of *P. esculentus *constitute one breeding system with different appearances under different ecological conditions. The gamete pattern hypothesis would suggest that the all-hybrid populations are a mosaic of intrinsically different variants of this breeding system. The latter would, in other words, suggest a breeding system with high biodiversity and various evolutionary significant units.

In this study, we first document the adult genotype frequencies over six years in a sample of 12 Swedish ponds to investigate whether different temporally stable population types exist. We also determined whether body length increases with R/L dosage effect from LLR through LR to LRR, or whether body length is larger in adult triploids (LLR and LRR) than diploids (LR).

Secondly, we test the selection hypothesis in adults by investigating LLR-, LR- and LRR-specific survival rates in each of the 12 ponds. Survival rates were estimated from mark-recapture data, and the effect of genotype, sex, time and season on survival was determined by model selection. Since genotype (LLR, LR, LRR) is not heritable (cf. Figure 1), survival is the only relevant measure of fitness in this system. If selection at the adult stage is responsible for a pond being dominated by one genotype, then the proportion and survival of each genotype in the 12 ponds should be positively correlated.

Thirdly, we test the selection hypothesis and the gamete pattern hypothesis simultaneously at juvenile stages in a subset of three ponds, i.e. the most extreme LLR-rich, LR-rich and LRR-rich ponds. Genotype proportions were assessed in a cohort of eggs, tadpoles, metamorphs and one year old froglets from each of the three ponds. If selection is responsible for one genotype being dominant among adults in a certain pond, this genotype is expected to rise in frequency during successive juvenile stages. Alternatively, if the gamete pattern is responsible for the adult genotype frequency, the genotype that is dominant among adults is expected to be dominant already in the egg stage.

## Results

### Adult genotype proportions

**Figure 2:**
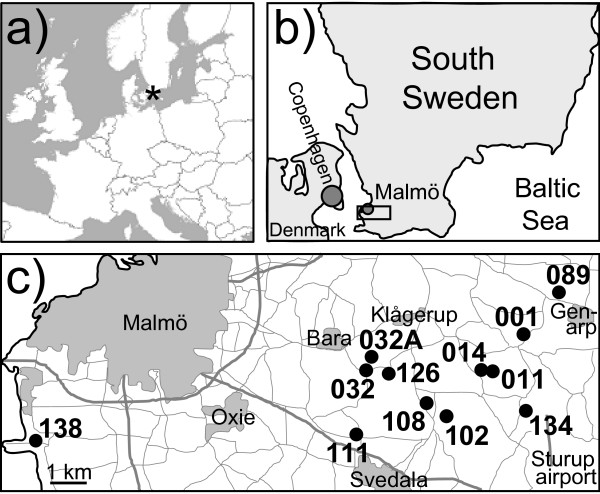
**Geographic location of the study**. a) South Sweden (star) in Western Europe b) the study area (rectangle) in South Sweden, and c) the 12 ponds within the study area.

Including recaptures, we caught 5051 LLR, LR and LRR frogs above 55 mm in the 12 Swedish ponds (Figure 2) during two annual catching rounds, 2002-2007 (listed in Additional file 1: Number of *P. esculentus *of various genotypes caught in different ponds and years). Among males, there were 42.6% LLR, 53.2% LR and 4.2% LRR; among females, 18.2% LLR, 45.0% LR and 36.9% LRR. The sex ratio in the sample was 40.4% males and 59.6% females, but this might be biased by differential behaviour and sampling effort (see methods). In addition, a total of six LL frogs were caught; no RR frogs were encountered. Four different individuals (caught a total of 7 times) were classified as LLRR by DNA flow cytometry. Excluding the six LL and the seven LLRR captures plus 27 captures of triploids with uncertain genotypes (see methods), the mean sample size per catching round per pond was 14.2 (range 2-43) for males and 20.9 (range 1-71) for females.

No overall seasonal effects were found between catching rounds one and two as tested over all ponds and years (paired t-tests: mLLR: t_71 _= 1.865, P = 0.066; mLR: t_71 _= -1.746, P = 0.085, mLRR: t_71 _= 0.067, P = 0.947; fLLR t_71 _= -0.742, P = 0.472; fLR t_71 _= 0.151, P = 0.880; fLRR t_71 _= 0.635, P = 0.528; Bonferroni-corrected α_4 _= 0.0125). This analysis might not reveal seasonal effects differing among ponds or years, but a detailed visual inspection of increases and decreases between catching rounds one and two revealed no patterns. In the following analyses, the first and second catching rounds were therefore pooled or used as replicates.

**Figure 3:**
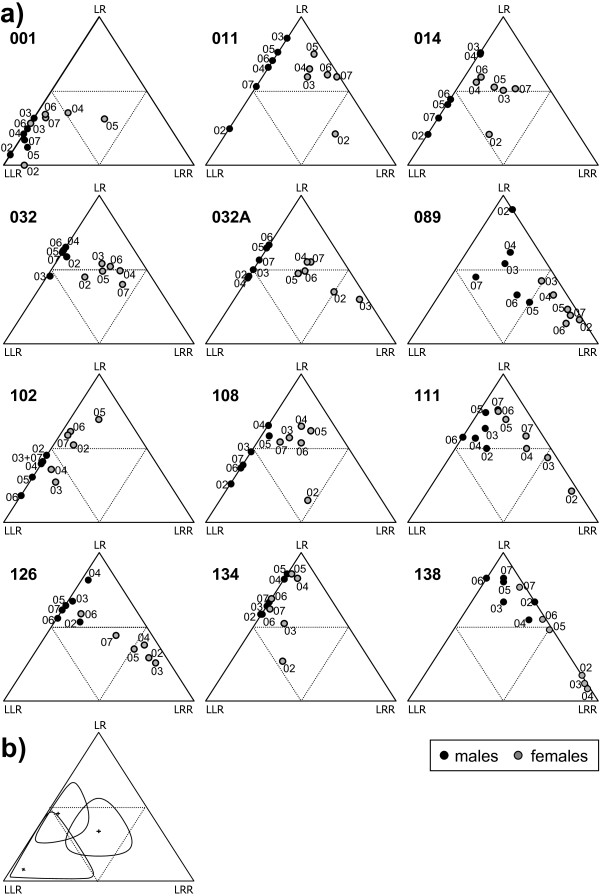
**Ternary plots of genotype compositions**. a) Proportions of LLR, LR and LRR among males and females of *P. esculentus *in 12 Swedish ponds (numbers 001-138) over six years (2002-2007). Each point represents the sum of two catching rounds per year; labels indicate the year (02-07). b) 95% confidence intervals around three fictive samples of 30 individuals with different genotype compositions.

The genotype proportions obtained in the 12 Swedish ponds over the six years are illustrated in Figure 3a. The two annual catching rounds were pooled to increase the sample sizes, which thus became mean 28.3 (range 6-64) for males and mean 41.8 (range 9-97) for females. As expected (see Background), LRR were rare among males, which is the main reason for the clear difference between male and female genotype proportions. By providing examples of 95% confidence intervals for similar sample sizes, Figure 3b suggests that most of the year-to-year variation observed within ponds and sex is due to sampling stochasticity. From inspection of Figure 3a it is evident that ponds 001, 011 and 089 were the most different ponds with non-overlapping genotype distributions for both sexes.

GLM (generalized linear model) analyses of genotype frequency on year and pond (both categorical) showed a highly significant effect of pond for all genotypes (Table 1). The effect of year was smaller, but nevertheless highly significant for most genotypes, especially LR and LRR females. In addition, the interaction of pond and year was significant for half of the genotypes.

**Table 1:** GLM with year and pond as categorical variables (n = 144 catches, df = 72).

	**Res**.	Year (df = 5)		Pond (df = 11)		Interaction (df = 55)	
	**dev**.	F	P	F	P	F	P
mLLR	90.87	5.57	0.00022***	20.41	< 2.20e-16 ***	1.56	0.03855
mLR	93.46	4.94	0.00060**	14.63	2.37e-14***	1.62	0.02812
mLRR	29.72	1.82	0.1197	64.40	< 2.20e-16***	2.74	3.34e-05 ***
fLLR	124.66	1.80	0.1238	20.39	< 2.00e-16***	1.20	0.2350
fLR	89.65	14.90	5.08e-10***	13.28	2.19e-13***	2.89	1.38e-05***
fLRR	82.81	18.38	1.02e-11***	46.49	< 2.20e-16***	4.54	1.92e-09***

* Stars indicate 0.05*, 0.01** and 0.001*** significance levels after Bonferroni-correction: α_4 _= 0.0125*, 0.0025** and 0.0003***. Res. dev = residual deviance.

**Figure 4:**
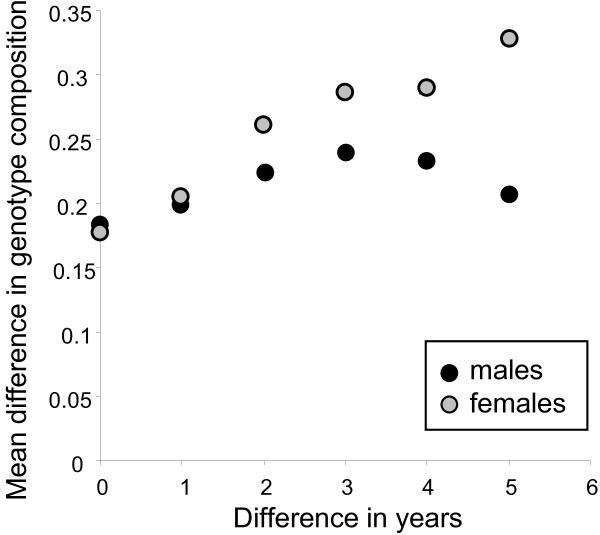
**The effect of time on changes in genotype composition**. Mean pairwise difference in genotype composition between catching rounds within ponds as a function of the difference in years between these catching rounds. The pairwise differences in genotype composition between catching rounds were measured as their distance in a ternary plot.

The mean difference in genotype composition among catching rounds, as measured by their distance in a ternary plot, was smallest between catching rounds from the same year (Figure 4). For males, the differences peaked or stabilized after three years, whereas for females the genotype differences continued to increase with time. As the pairwise means are not independent, the trends of Figure 4 cannot be tested by correlation or regression. Instead, a Mantel test with permutation would be needed. However, Mantel test programmes do usually not take missing values, and the present data set had many missing values, because between-pond comparisons would have been meaningless.

**Figure 5:**
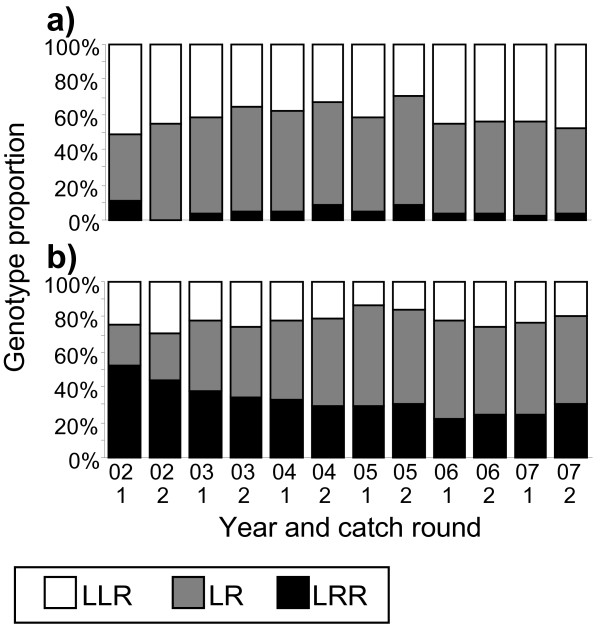
**Temporal development of genotype proportions summed over the 12 Swedish ponds**. a) males and b) females. The ponds were sampled twice per year from 2002 to 2007.

The year effect in LR and LRR females (Table 1) and the increasing female genotype composition differences over time (Figure 4) both reflect the fact that the proportion of LR females increased during the study period at the expense of LRR females (Figure 5). Inspection of Figure 3 also reveals that only pond 032 did not show a net increase in LR females between 2002 and 2007.

### Adult body lengths

Adult body length was measured in 4968 frogs and differed highly significantly between sexes, genotypes, ponds and all their interactions (ANOVA, F_4900 _ranged from 54.92 to 2060.44, P < 2.2e-16, for the three main effects, and F_4900 _ranged from 2.08 to 5. 54, P from 0.0056 to 5.73e-13, for the interactions). Mean length increased in the order mLLR, mLR, mLRR, fLLR, fLR, fLRR with means ± S.D. of 64.5 ± 4.6, 66.0 ± 5.7, 69.1 ± 5.7, 72.3 ± 7.1, 74.4 ± 9.0 and 75.4 ± 9.7 mm, respectively. Thus, males were smaller than females, as is usual in anurans, and clearly LLR < LR < LRR within sexes, indicating genome dosage effect. The means cover large variation within sexes and genotypes, as the frogs grow throughout their life.

### Adult survival and the selection hypothesis

Of the 5051 adult LLR, LR and LRR frogs caught over the six years, 1011 (20.0%) were recaptures. Ninety (1.8%) of the captured individuals (mostly males) were killed by us by accident or for crossing experiments. These were coded accordingly in the MARK input file, so that their removal did not affect the survival estimates. A total of 2.8% of the previously toe-cut, recaptured frogs had lost their transponder. Only in ponds 089, 111 and 138 were LRR males sufficiently frequent to include recaptured individuals; from the remaining ponds, LRR males had to be excluded from the data set.

For testing the selection hypothesis in adults, adult survival rates (Φ) were estimated in Comack-Jolly-Seber mark recapture models. Goodness of fit tests (in U-care) showed no overdispersion (ĉ = 0.21-0.46). Only pond 001 was significant for transience (P = 0.017); no ponds were significant for trap-dependence. The data were thus in fairly good accordance with the assumptions of the Comack-Jolly-Seber model.

Twelve combinations of the parameters genotype, sex, time and season (nested within time) were used to model both recapture probability (p, necessary for estimating survival) and survival (Φ). For recapture probability, eight ponds had just one best model whereas four ponds had two good models with similar fits (ΔAICc > 2). All ponds had a clear distinction in ΔAICc between the best one or two models and the poorer models. Overall, time was the most important parameter for recapture probability, as six of the 12 ponds had time as their best model (see Additional file 2: AICc weights for different models of p in the 12 ponds keeping Φ constant). This reflects that sample sizes often differed over time. Season, genotype and/or sex were of highest importance in the remaining six ponds, indicating that although these factors might not have high general importance, they sometimes had high local importance. Combinations of time and other factors were not favoured. This was not surprising, since time was highly parameterized, and AIC model selection favours models that fit the data well with few parameters.

For survival, the combination of genotype, sex and time gave the best fit in eight of the 12 ponds (see Additional file 3: AICc weights for different models of Φ in the 12 ponds using previously identified best models for p). For two ponds (032 and 102) this highly parametized model did not converge and, instead, the constant model was favoured. For only two ponds (014 and 134) the model with genotype, sex and time was clearly rejected; instead, models with season and constant survival, respectively, gave the best fits.

Adult yearly survival estimates averaged 0.31 over all 12 Swedish ponds, ranging from 0.17 in pond 134 to 0.46 in pond 001 (Table 2). This average translated into a mean life span of less than 11 months as adults (lifespan = 1/-ln(survival) [22]). However, survival varied among genotypes, sexes and ponds and the ponds varied with respect to which genotypes and sex had the higher survival.

**Table 2:** Yearly survival estimated for the six genotypes in 12 ponds.

Pond	mLLR	mLR	mLRR	fzLLR	fLR	fLRR	Genotype mean
001	0.489	0.478		0.458	0.450	0.443	0.464
011	0.170	0.312		0.196	0.329	0.249	0.251
014	0.439	0.451		0.454	0.470	0.449	0.453
032	0.311	0.334		0.302	0.320	0.329	0.319
032A	0.192	0.261		0.208	0.265	0.215	0.228
089	0.440	0.437	0.444	0.365	0.368	0.369	0.404
102	0.265	0.272		0.286	0.298	0.272	0.278
108	0.293	0.165		0.398	0.224	0.077	0.232
111	0.272	0.265	0.281	0.217	0.263	0.261	0.260
126	0.281	0.268		0.297	0.279	0.308	0.287
134	0.148	0.189		0.175	0.158	0.176	0.169
138	0.347	0.624	0.564	0.272	0.578	0.047	0.406

Pond mean	0.304	0.338	0.430	0.302	0.334	0.266	0.312^1^

^1 ^Mean of pond means; not of genotype means.

With ponds as replicates, there were no significant correlations between the mean proportion of a genotype (within sex) and its estimated relative (within pond) survival rate over the six year period (Spearman rank correlation tests: mLLR: rho_12 _= 0.245, P = 0.437; mLR: rho_12 _= 0.508, P = 0.920; fLLR: rho_12 _= 0.664, P = 0.021; fLR: rho_12 _= 0.252, P = 0.424; fLRR: rho_12 _= -0.203, P = 0.528; Bonferroni-corrected α_5 _= 0.010; LRR males could not be tested as survival data were obtained from three ponds only). The study did thus not provide evidence for the survival hypothesis which predicts that differences in survival among genotypes produce the differences in adult genotype composition observed among ponds.

### Offspring genotype proportions and both hypotheses

**Figure 6:**
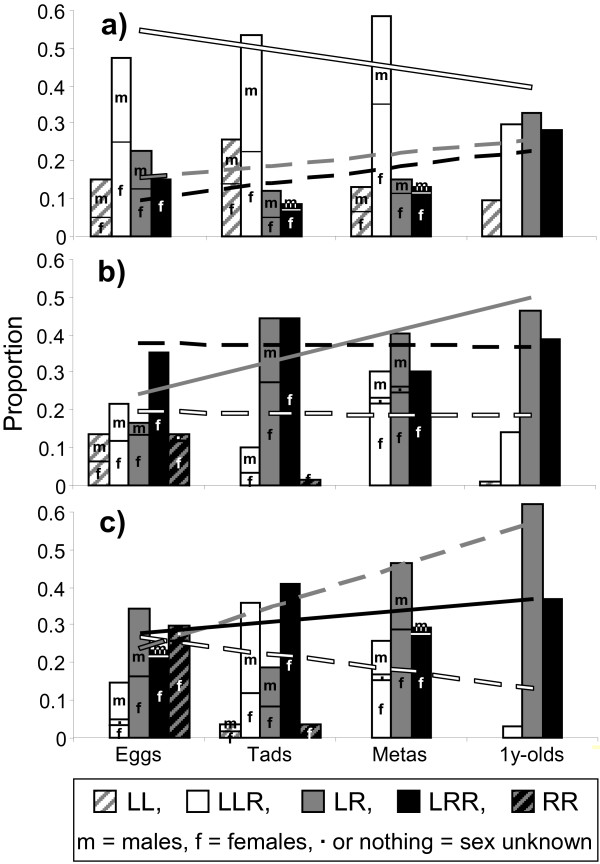
**Genotype distributions at four stages of the 2006 cohort in three ponds**. a) pond 001 (LLR-rich), b) pond 011 (LR-rich) and c) pond 089 (LRR-rich). The four stages are eggs, tadpoles, metamorphs and one year-olds. Regression lines for LLR, LR and LRR are added for comparison of slopes and intercepts. The line is drawn solid for the genotype that is dominant among adults in the particular pond; the lines for the remaining genotypes are dashed.

The genotype distribution of the offspring sampled is shown in Figure 6. A GLM was fitted for each genotype, LLR, LR and LRR (residual deviance = 81, 73 and 20 for LLR, LR and LRR respectively, with 27 df). Two of the three GLMs were significant for pond (LLR: t_27 _= 8.51, P = 0.003*; LR: t_27 _= 3.65, P = 0.047; LRR: t_27 _= 10.03, P = 0.001*; Bonferroni-corrected α_2 _= 0.025), but there was neither significance for stage nor for the interaction between pond and stage (data not shown).

Inspection of the regression parameters showed that in the LLR model, the intercept for pond 001 was almost significantly higher than that for pond 011 (t_18 _= -2.390, P = 0.028, Bonferroni-corrected α_2 _= 0.025), and in the LRR model, the intercept for pond 089 was significantly higher than that for pond 001 (t_18 _= -2.582, P = 0.019; Bonferroni-corrected α_2 _= 0.025). The remaining four intercepts and all six slopes did not differ significantly within genotypes between ponds (data not shown). As positive differences in slopes would support the selection hypothesis and positive differences in intercepts would support the gamete pattern hypothesis (see methods), this analysis thus provided no support for the selection hypothesis and only very weak support for the gamete pattern hypothesis. The offspring study had relatively low discriminative power because the genotype proportions in one year-olds only incompletely matched the mean genotype proportions of adults.

## Discussion

The present study is the first to thoroughly investigate the temporal stability of all-hybrid populations consisting of LLR, LR and LRR frogs. Genotype proportions varied more among ponds than among years. Thus, especially ponds with extreme adult genotype proportions retained their differences over the six-year study period. The largest temporal change was an increase in LR females that occurred in most ponds in parallel, thus not diminishing pond differences. Systematic seasonal changes were absent.

The genotype composition differences among ponds could not be attributed to pond-specific selection regimes (selection hypothesis), neither in adults nor offspring. The alternative gamete pattern hypothesis was not investigated in adults in the present study, whereas the offspring study provided only very weak support for it. In the following, we will first discuss the data on body size, sex ratio and survival obtained in the present study and estimate how many generations the study period spanned. Then we will discuss problems of distinguishing between the selection and gamete pattern hypotheses, identify potential advantages and disadvantages of differential selection for the all-hybrid populations and, finally, briefly review the water frog literature on this topic.

### Body size, sex ratio, survival and generation time

As adult body size constitutes an important phenotypic difference between *P. lessonae *and *P. ridibundus *with ecological implications, knowing the relative body sizes of LLR, LR and LRR can be of importance for predicting their fitness in different habitats. In the present study, a dosage effect pattern was observed among adults so that LLR < LR < LRR within both sexes, in line with *P. lessonae *being smaller and *P. ridibundus *being larger than *P. esculentus *e.g. [12]. Thus, although triploid frogs have larger cells [7,21], adult triploid LLR frogs were not larger than diploid LR frogs. This is in line with the general observation that in vertebrates polyploidy does not imply increased body size [23]. Thus, although most triploids start life larger than diploids because they usually derive from larger, diploid eggs [10,24], this initial difference disappears. Dosage effect also applies to other phenotypic features, such as the metatarsal tubercle (callus internus) size and tibia length [7,18-20]. The callus internus is important for digging in the partially terrestrial *P. lessonae*, while leg length is more important in the more aquatic *P. ridibundus *[19]. In phenotypic and ecologic contexts, LLR and LRR are thus more different from each other than from LR, so that summarizing them as "triploids" is not informative in these contexts.

The sex ratio in *P. esculentus *is of interest because the two sexes are not expected to be produced in equal numbers (cf Figure 1). However, empirical data on sex ratio are difficult to obtain because the observed sex ratio in field samples could have been influenced by the differential distribution of the two sexes within ponds at different times of the year and males being easier to catch from mid May to late June when they call. Thus, samples were thought to be consistently male-biased in a study of Hotz et al. [25]. In contrast, the present attempt to catch at least 10 of each sex should have led to overestimation of the rarer sex, which was males. The overall 59.6% females found in the present study among adults is thus an underestimate. Offspring sex ratios should be less prone to sampling bias, as sex-specific behaviour is not expected in eggs, tadpoles and metamorphs, and the sexes could not be distinguished during catching (male and female tadpoles of *P. ridibundus *were not found to differ in larval period or weight at metamorphosis [26]). Furthermore, offspring sex ratios should closely reflect initial sex ratio. The 68.1% females encountered among offspring with hybrid genotypes and sexed in the present study may thus represent the best estimate of sex ratio in this system so far. This figure fairly well matches results from modelling all-hybrid populations based on gamete production by the various genotypes predicting 65.0% females (in both "normal" and LRR-rich populations [14]). Also in the much more widespread L-E system (*lessonae*-*esculentus *system), *P. esculentus *have an expected [27] and observed (61% [28]) female-biased sex ratio. In the L-E system, *P. esculentus *is usually only diploid LR and always make clonal R gametes. As a consequence, the hybrids are dependent on mating with a *P. lessonae *to produce new hybrids. In an organism like *P. esculentus *where few males are needed to satisfy the mating requirements of many females, female bias has the advantage of reducing the two-fold cost of sex experienced by normal sexual species.

The mean survival of 0.31 per year found for *P. esculentus *in this study appears rather low compared to 0.61 (2 ponds, 5 and 7 years, respectively [29]) and 0.53-0.70 (4 ponds, 4 years [30]) for Swiss L-E system *P. esculentus*. Both studies also analyzed mark-recapture studies with the MARK programme, but the latter also modelled migration, increasing the survival estimates. Survivals around 0.3 have, however, also been reported from tree frogs in southern Germany and Switzerland [31,32].

For interpreting the results on temporal stability, it is relevant how many generations the six year study period spanned. In Scandinavia, males are sexually mature when two to three years old, whereas females usually need three years to mature [19]. A rough average would be 2.75 years. The mean adult life span was here estimated to 11 months = 0.92 years, so the midpoint of the reproductive period should be around 2.75 + 0.5*0.92 = 3.2 years. This might be a low estimate of generation time, as female fecundity increases with body length, i.e. with age. The six year study period thus probably covered between one and a half and two generations. Even longer studies would be needed to investigate long-term development of genotype proportions in all-hybrid populations. However, the general instability of pond habitats may interfere with questions about long-term stability of frog populations.

### Differential selection

The lack of evidence for the selection hypothesis in the adult survival study can be interpreted in at least two ways. By one interpretation, very small survival differences are sufficient for producing the genotype proportions observed, and thus a larger number of ponds with extreme genotype proportions would be necessary for obtaining a significant correlation. Alternatively, pond variation in differential selection on genotypes is not important - at least not at the adult stage. The offspring study was better suited for detecting pond variation in differential selection, because the selection potential is much larger at early stages that exhibit higher mortality. Moreover, the offspring study had the advantage of testing both hypotheses simultaneously. The weak outcome was most probably due to methodological difficulties in obtaining representative samples of the various life stages. Genotypes might distribute themselves non-randomly in space and time during spawning, larval development, metamorphosis, and/or as one year-olds and adults. Our effort to distribute sampling over the entire pond and most of the period where each stage was available might not have sufficed for obtaining random samples.

The reproductive dependence of all genotypes in the all-hybrid populations (LLR, LR and LRR) upon each other means that differential selection is not required for coexistence of the three genotypes. However, this does not exclude that differential selection could promote differences in genotype proportions among ponds. Differential selection could potentially have both advantages and disadvantages for the all-hybrid populations. One advantage is that niche-based coexistence can confer increased carrying capacity [4,33,34]. On the other hand, differential selection could imply the disadvantage of increased hybrid load in all-hybrid populations with extreme environments. Hybrid load should increase because extreme population compositions biased toward one genotype should increase the production of lethal non-hybrid genotypes, unless the gamete pattern is changed. Based on these considerations, it cannot be predicted whether differential selection should occur in all-hybrid populations of *P. esculentus*.

In other water frog breeding systems, various studies have tested one or more of the four hybrid models presented in the introduction. These studies were done in the L-E (*lessonae*-*esculentus*) system, and one study [13] also included the very similar *perezi-grafi *system. This latter study found significant habitat differentiation among the three water frog species (*P. lessonae*, *P. ridibundus*, *P. perezi*) and their two hybrids (*P. esculentus *and *P. grafi*) in support of a mosaic hybrid zone with bounded hybrid superiority, i.e. in support of differential selection. Three studies aiming to investigate the relative importance of the frozen niche variation and the general purpose genotype models all concluded that both models may apply [25,35,36]. Finally, one study provided evidence for frozen niche variation by showing that clone mixtures of tadpoles had higher survival than monocultures [37]. Thus all studies suggest an importance of niche differentiation/differential selection in shaping the composition of hybrid populations, although the frozen niche variation model was not better supported than the non-niche-based general purpose model.

## Conclusions

Valuable data on genotype-specific body lengths, adult survival, sex ratios and temporal genotype proportion stability in all-hybrid populations of *P. esculentus *were obtained. A role of differential selection in shaping genotype proportions was neither identified in the adult, nor in the offspring study. With only weak evidence for alternative processes shaping genotype proportions, the selection hypothesis can, however, not confidently be rejected. In spite of multiple approaches, the importance of differential selection in shaping genotype proportions thus remains an interesting, but largely unsettled, matter in all-hybrid populations as well as in other water frog breeding systems.

## Methods

### Adult sampling

The study was performed in 12 ponds in Skåne (Scania), Southern Sweden (Figure 2, coordinates in [7]). In each of the 12 ponds, a sample of adult frogs was caught twice per year, with catching dates differing among ponds and years (dates listed in Additional file 1: Number of *P. esculentus *of various genotypes caught in different ponds and years). The frogs were caught at night by hand or dip net, dazzling them with a torch and moving about with waders and sometimes a small rubber boat. Especially in later years, an effort was made to obtain at least thirty adults per catching round, including at least ten individuals of each sex. To obtain these numbers, catching rounds could extend over several days (or, rarer, weeks); the catching date was then calculated as the mean date on which the frogs were caught. Due to removal of floating vegetation, the number of frogs in pond 014 was so low in 2006 and 2007 that additional sampling in the 10 m distant neighbouring pond was necessary for reaching reasonable sample sizes.

The frogs were brought to the nearby Stensoffa Field Station, Torna Hällestad, for processing. New frogs were measured from snout to vent (spine straight) with a slide calliper, were individually marked with a PIT tag transponder (Trovan ID101, Euro I.D., DE) and had a toe-tip cut off for microsatellite genotyping. In 2002-2004 also a blood sample was taken for genotyping by DNA flow cytometry. Recaptured frogs were just measured and identified via the PIT tag. All frogs were returned to their source pond within a few days of sampling, with the exception of minor numbers used in crossing experiments in 2002, 2004 and 2006.

Adult frogs were defined as individuals with at least 55 mm from snout to vent. Permits for catching, toe-clipping and marking frogs were obtained from the Swedish authorities (Länsstyreslen I Skåne Län 522-18591-02, 522-9286-03, 522-6571-04, 522-10481-05 and Djurskyddsmyndigheten M62-05).

### Offspring sampling

Ponds 001, 011 and 089 had the most divergent genotype distributions, and were therefore picked for the study of juvenile stages. In 2006, these three ponds were sampled for eggs, tadpoles and metamorphs and in 2007 for one year-olds as judged by their size.

Eggs were sampled 5-30 June 2006; no eggs were found outside this period. Mating pairs lay several egg clutches in rapid succession and the fertilized eggs within these clutches often differ in genotype. To obtain random egg samples, the ponds were searched for new egg clutches every three days. From every clutch judged by location, egg sizes and age to be from a different pair, approximately 20 eggs were sampled and the remaining part of the clutch was marked. The eggs were brought to the field station. Upon reaching the free-swimming feeding stage, one healthy-looking tadpole per clutch was randomly chosen for rearing to metamorphosis, whereas the remaining tadpoles were returned to their source pond. Healthy-looking tadpoles were preferred for rearing because abnormal tadpoles were assumed to die under natural conditions and do thus not provide information on population maintenance. The chosen tadpoles were reared in outdoors 40-liter tubs with up to 15 tadpoles per tub and food ad libitum (as described in [9]). In total, tadpoles were reared from 44, 61 and 65 egg clutches from ponds 001, 011 and 089, respectively, and DNA data were obtained from 95% of them, i.e. from 40, 60, and 62 individuals, respectively. Sex was determined by dissection approximately a week after tail resorption (as described in [14]). For statistical analysis, the eggs were divided into two equal-sized groups according to sampling date: sample 1 constituted the earlier collected eggs and sample 2 the later collected eggs.

Tadpoles were sampled by dip-netting; 30 tadpoles on 6 July 2006 (sample 1) and another 30 on 20 July 2006 (sample 2) in each of the three ponds. On 20 July the tadpoles showed large size variation, but intermediate-sized tadpoles were preferred. The tadpoles were reared and analyzed as described for the eggs. Of the 60 tadpoles sampled in each pond, DNA samples were obtained from 58, 59, and 59 (97%), respectively.

Metamorphs were sampled on 1, 6, 11, 16, 21 and 31 August 2006. On each of these dates, 10 metamorphs with a few millimetres of unresorbed tail were sampled in each of the three ponds. The metamorphs were all sexed and DNA-analyzed after ten days of rearing. For statistical analysis, the metamorphs from the first three dates were pooled and labelled sample 1 whereas those from the last three dates constituted sample 2.

One year-olds were sampled in 2007. Per pond, 30 were sampled in May (sample 1) and 30 in July (sample 2). As one year-olds were not nearly as numerous as offspring at the earlier stages, they were not sacrificed and sexed, but were released to their source pond after removal of one or two toes for DNA analysis.

Permits for catching, rearing and killing juveniles were obtained from the Swedish authorities (Länsstyreslen I Skåne Län 522-10481-05 and Djurskyddsmyndigheten M62-05).

### Genotyping

The adult frogs from 2002-2004 were caught and genotyped using a combination of microsatellite analysis and DNA flow cytometry as described in [7]. The adults and offspring from 2005-2007 were genotyped using microsatellite analysis of four loci with dosage effect, capable of distinguishing LL, LLR, LR and LRR and RR, as described in [9,14,18]. General agreement between the two genotyping protocols was confirmed in frogs analyzed with both protocols because they were caught in both time periods.

Some frogs had mixed genotypes where microsatellite loci and/or flow cytometry analyses disagreed on the genotype. As the resolution for identifying mixed genotypes varied with the methods applied, no analyses on mixed genotypes were possible. The 45 frogs with mixed genotypes from 2005-2007 were assigned to LLR, LR or LRR according to the majority of the loci analyzed (sometimes more than the four dosage effect loci were analyzed). Likewise, 23 frogs from 2002-2004 recorded to have mixed diploid genotypes were included in the data set as LR. However, 26 frogs from 2002-2004 recorded to have mixed or mosaic triploid genotypes were excluded from the data set, as it was often unsure whether they had most resemblance with LLR or LRR.

### Analysis of adult genotype proportions and lengths

First, it was tested whether genotype proportions differed systematically between the first and the second catching round per year. If not, the two catching rounds per year could be pooled to increase sample sizes, or used as replicates.

Ternary plots were used for displaying the proportion data for three genotypes (LLR, LR and LRR) in two dimensions. The ternary plots were drawn in the programme Past, version 1.80 [38]. Ternary confidence areas were drawn with programmes provided by Gert Jan Weltje [39] and the software Grapher (version 7, Golden Software Inc, Golden, Colorado, USA).

In the ternary plots and the following statistical analyses of adult genotype proportions, males and females were treated separately, because our sampling did not necessarily reflect the natural sex ratio. Sometimes our sampling might have been biased by the different behaviour of the two sexes; at other times we biased the sample sex ratio ourselves in order to obtain at least ten individuals of the rarer sex.

For investigating whether different temporally stable population types exist, the effects of pond and year on genotype proportions were analyzed. Year was treated as a discrete variable, because genotype proportions were expected to fluctuate over time. A continuous, temporal change in the proportion of a genotype is not meaningful, since it would imply that genotype proportions might eventually reach or extend beyond zero or one. The data were analyzed with generalized linear models (GLMs) in the programme R version 2.8.0 [40]. GLMs with binomial error distributions (logit) have the advantage of coping with proportion data in a way where sample sizes are taken into account. Pooling the two annual catching rounds to avoid very small samples was therefore not necessary. Using a binomial error distribution requires binomial data, as for example "LLR male" and "non-LRR male". Therefore, a model was fitted separately to each genotype within sex (mLLR, mLR, mLRR within males and fLLR, fLR, fLRR within females). For most models, the data exhibited overdispersion (residual deviation > the degrees of freedom), wherefore quasibinomial error distributions and F tests were used. Fitting a model to all three genotypes (LLR, LR and LRR) within each sex is statistically redundant, because the genotype proportions add up to one and the result for the last genotype is therefore given by the first two analyses. Nevertheless, to facilitate reading and interpretation, tests for all three genotypes are provided in the results tables for this and subsequent analyses. However, when Bonferroni-correcting the significance level, α, the apparently three tests for LLR, LR and LRR only count as two; thus tests for mLLR, mLR, mLRR, fLLR, fLR and fLRR count as four tests. Strict Bonferroni corrections were used, because sequential Bonferroni corrections would differ according to which two tests are considered redundant.

Differences in adult body length as a function of sex, genotype, pond and their interactions were analyzed with an ANOVA in R [40].

### Testing the selection hypothesis in adults

The selection hypothesis implies that the proportion of each genotype in a pond is affected by its relative survival. For testing the hypotheses in adults, survival probabilities therefore had to be estimated from the six year's mark-recapture data and correlated with the genotype proportion data presented above.

Adult survival rates were estimated using the Comack-Jolly-Seber mark-recapture model. The ponds had to be analyzed separately, because their catching dates, and thus time intervals between catching rounds, differed. These time intervals were expressed in fractions of years so that the output survival estimates were in units of years.

The Comack-Jolly-Seber model assumes that all marked individuals within predefined groups (here mLLR, mLR, mLRR, fLLR, fLR and fLRR) have the same probability of recapture and of survival, that marks are not lost and that sampling events are short compared to the time intervals between samplings [22]. First, the goodness of fit of the data to the assumptions was tested in U-care vers. 2.02 [41,42]. Included in this programme are tests for transience (animals migrating through the population) and trap-dependence (animals liking or avoiding capture).

Models were constructed and selected in the programme MARK [43]. MARK estimates Φ = survival rate and p = recapture probability - each as a function of parameters of interests. A set of candidate models are evaluated by AICc according to how well they minimize deviance from the data with the fewest parameters possible [22]. In the present study, the full model was Φ(genotype*sex*time) p(genotype*sex*time), where genotype was LLR, LR or LRR, sex was male or female and time was time-dependence; i.e. varying survival over the 11 time intervals between the 12 catching rounds. The remaining candidate models were reduced versions of this full model. Some candidate models included season, as a reduced alternative to time. The season parameter implied difference in survival in the summer period between within-year catching rounds as opposed to the period between catching round two in one year and catching round one the following year. A model assuming constant parameters, symbolized by a dot, was also included among the candidate models for p and Φ.

The analysis was done in three steps. First, the best models for recapture probability were selected for every pond. This was done by keeping Φ constant while evaluating different models for p. All models with ΔAICc below two, and thus with the best fits, were selected for the next steps of the analysis. Secondly, the best model(s) for p were inserted into the candidate models estimating survival (Φ), and these survival models were evaluated using AICc. Finally, mean yearly survival rates over the six years were calculated by averaging over all the survival models that did not contain time or season, weighed by the AICc weight by each model.

Testing for the selection hypothesis among adults implied testing for correlation between the proportion (within sex) and relative survival within each genotype in R [40]. Spearman rank correlations were used because proportion data have non-normally distributed residuals and were furthermore highly overdispersed in a GLM with genotype proportion as dependent variable and relative survival as independent variable. Relative survival rates for mLLR, mLR, (mLRR,) fLLR, fLR, and fLRR were calculated as their difference from the pond mean. These relative survival rates were preferred to absolute survival rates because pond differences in absolute survival could otherwise blur the correlation.

### Testing both hypotheses in juvenile stages

The analysis of juvenile stages was made to investigate how pond differences in adult genotype composition arise, i.e. why pond 001 have more LLR, pond 011 more LR and pond 089 more LRR when compared to each other. The selection hypothesis predicts that the differences arise by differential selection, so that for example in pond 001, the proportion of LLR increases more during subsequent juvenile stages than in the other ponds. Thus, a significantly higher slope for LLR in pond one as compared to ponds 011 and 089 would support the selection hypothesis, and similarly for the slopes of LR in pond 011 and LRR in pond 089. In contrast, the gamete pattern hypothesis predicts that the differences in adult genotype compositions among ponds are a result of differential gamete production by the same genotypes in different ponds. In pond 001 a high proportion of LLR should thus be present already in the egg stage of new generations. Thus a significantly higher intercept of LLR in pond 001 than in ponds 011 and 089 would support the gamete pattern hypothesis, and similarly for the intercepts of LR in pond 011 and of LRR in pond 089.

To test for such differences in slopes and intercepts, three GLMs with quasibinomial error distributions were fitted; one for LLR, one for LR and one for LRR (though the last was redundant). In the LLR model, pond 001 was first (used as intercept), in the LR model pond 011 was first, and in the LRR model pond 089 was first. Males and females were pooled within genotypes, as their proportions were not expected to be biased by behaviour and sampling technique, and sex data were not available for the one year-olds. As for adults, quasibinomial error distributions were used. Stage (eggs, tadpoles, metamorphs, one year-olds) was coded as a continuous variable (0, 1, 2, 3) with eggs as stage zero so that the fitted value at this stage would be the intercept. Pond was treated as a discrete variable.

## Authors' contributions

HUR acquired the funding and supervised the project. HUR, CJ and MA designed the mark-recapture study of adults; CJ and MA caught and genotyped adults 2002-2004 whereas DGC caught and genotyped adults 2005-2007. DGC planned and conducted the offspring study. SR participated in planning the microsatellite analyses and carried out most of the laboratory work. DGC undertook the statistical analyses and wrote the article. All authors read and approved the final manuscript.

## Supplementary Material

Additional file 1 **Number of *P. esculentus *of various genotypes caught in different ponds and years**. Complete table of frogs captured. Click here for file

Additional file 2 **AICc weights for different models of p in the 12 ponds keeping Φ constant**. Table of AICc weights. Click here for file

Additional file 3 **AICc weights for different models of Φ in the 12 ponds using previously identified best models for p**. Table of AICc weights. Click here for file
