# Supplementary material for: Coexistence of diploid and triploid hybrid water frogs: population differences persist in the apparent absence of differential survival
Source: BMC Ecol. 2010 May 27;10:14. doi: 10.1186/1472-6785-10-14 (PMC2902419; doi:10.1186/1472-6785-10-14)
Supplement: Additional file 1 — Number of P. esculentus of various genotypes caught in different ponds and years. Complete table of frogs captured. [file 1472-6785-10-14-S1.PDF]

Additional file 1. Number of *P. esculentus* of various genotypes (LLR, LR and LRR) caught in different ponds and years. m = male, f = female, n = total, catch = catching round.

Pond	Year	Catch	Date	Day	mLLR	mLR	mLRR	mn	fLLR	fLR	fLRR	fn	n
001	2002	1	14.05.02	14	15	1	0	16	2	0	1	3	19
001	2002	2	07.07.02	68	11	1	0	12	6	0	0	6	18
001	2003	1	22.05.03	22	9	4	0	13	5	1	0	6	19
001	2003	2	09.07.03	70	6	3	0	9	10	5	0	15	24
001	2004	1	16-18.05.04	16	25	6	0	31	6	6	3	15	46
001	2004	2	23.07.04	84	8	3	0	11	9	5	2	16	27
001	2005	1	16-22.05.05	21	15	2	0	17	2	4	8	14	31
001	2005	2	07-08.08.05	100	11	2	2	15	8	6	4	18	33
001	2006	1	14-28.05.06	22	20	9	0	29	9	9	1	19	48
001	2006	2	11.07.06	72	16	3	0	19	16	5	1	22	41
001	2007	1	25.05.07	25	21	3	0	24	8	8	0	16	40
001	2007	2	11-13.07.07	73	12	4	1	17	13	3	2	18	35
011	2002	1	22.05.02	22	6	2	0	8	1	4	7	12	20
011	2002	2	08.06.02	39	6	2	0	8	5	2	9	16	24
011	2003	1	20.05.03	20	2	8	0	10	4	19	7	30	40
011	2003	2	07.07.03	68	3	22	0	25	4	9	4	17	42
011	2004	1	15-19.05.04	16	7	31	0	38	2	26	9	37	75
011	2004	2	26.06.04	57	18	18	0	36	4	4	1	9	45
011	2005	1	12.05.05	12	6	17	0	23	0	11	2	13	36
011	2005	2	08.08.05	100	1	5	0	6	2	16	5	23	29
011	2006	1	12-28.05.06	22	6	13	0	19	2	16	6	24	43
011	2006	2	05-08.07.06	67	6	16	0	22	0	6	6	12	34
011	2007	1	22.05.07	22	7	12	0	19	0	17	7	24	43
011	2007	2	07-13.07.07	72	9	6	0	15	1	15	14	30	45
014	2002	1	23.05.02	23	5	3	0	8	7	4	2	13	21
014	2002	2	24.06.02	55	6	0	0	6	6	2	7	15	21
014	2003	1	21.05.03	21	2	14	0	16	2	13	8	23	39
014	2003	2	28.06.03	59	5	8	0	13	10	12	4	26	39
014	2004	1	25.05.04	25	5	16	0	21	4	12	1	17	38
014	2004	2	26.06.04	57	3	9	0	12	10	10	2	22	34
014	2005	1	13.-14.05.05	13	14	5	0	19	2	9	1	12	31
014	2005	2	08.08.05	100	3	7	0	10	7	8	5	20	30
014	2006	1	12.05.-10.06.06	39	12	9	0	21	4	12	1	17	38
014	2006	2	12.07.06	73	4	4	0	8	7	9	2	18	26
014	2007	1	21.05.07	21	16	9	0	25	3	6	3	12	37
014	2007	2	14.-16.07.07	76	3	0	0	3	1	6	4	11	14
032	2002	1	25.05.02	25	4	4	0	8	3	5	4	12	20
032	2002	2	13.07.02	74	7	13	1	21	4	4	0	8	29
032	2003	1	17.05.03	17	12	9	0	21	1	8	4	13	34
032	2003	2	16.06.03	47	22	20	1	43	7	12	5	24	67
032	2004	1	01.06.04	32	10	14	0	24	4	7	7	18	42
032	2004	2	14.07.04	75	5	14	0	19	2	13	8	23	42
032	2005	1	23.-25.05.05	24	11	21	0	32	10	14	8	32	64
032	2005	2	06.08.05	98	5	6	0	11	2	11	6	19	30
032	2006	1	13.05-05.06.06	32	5	8	0	13	4	14	6	24	37
032	2006	2	17-20.06.06	79	6	10	0	16	6	14	10	30	46
032	2007	1	29-30.05.07	30	4	11	0	15	3	9	9	21	36
032	2007	2	09.-10.07.07	70	6	5	0	11	5	9	10	24	35

032A	2002	1	13.07.02	74	3	2	0	5	5	7	11	23	28
032A	2002	2	16.07.02	77	3	3	0	6	1	7	9	17	23
032A	2003	1	17.05-04.06.03	33	3	2	0	5	1	6	16	23	28
032A	2003	2	22.06.03	53	2	3	0	5	1	8	15	24	29
032A	2004	1	31.05.04	31	4	0	0	4	3	7	3	13	17
032A	2004	2	15.07.04	76	6	8	0	14	3	10	5	18	32
032A	2005	1	25.-26.05.05	25	1	3	0	4	6	13	2	21	25
032A	2005	2	06.08.05	98	4	6	0	10	5	6	8	19	29
032A	2006	1	13.05-05.06.06	25	0	2	0	2	4	8	5	17	19
032A	2006	2	18.06.06	79	6	10	0	16	7	15	8	30	46
032A	2007	1	29.05.07	29	5	4	0	9	6	19	5	30	39
032A	2007	2	04.07.07	65	5	9	0	14	4	13	11	28	42
089	2002	1	11.-18.05.02	29	0	3	1	4	0	7	37	44	48
089	2002	2	08.07.02	69	0	6	0	6	1	2	9	12	18
089	2003	1	09.05.03	37	6	19	4	29	6	20	32	58	87
089	2003	2	13.07.03	74	5	8	8	21	2	20	14	36	57
089	2004	1	17.05.04	17	4	18	5	27	2	6	20	28	55
089	2004	2	22.06.04	53	2	6	4	12	2	12	13	27	39
089	2005	1	12.05.05	12	2	4	5	11	1	4	17	22	33
089	2005	2	03.08.05	95	2	1	4	7	1	7	17	25	32
089	2006	1	12.-28.05.06	18	6	8	11	25	5	9	44	58	83
089	2006	2	08.-09.07.06	69	7	8	9	24	4	3	23	30	54
089	2007	1	21.05.07	21	13	8	2	23	3	5	22	30	53
089	2007	2	10.-13.07.07	71	3	9	3	15	0	7	25	32	47
102	2002	1	26.06.02	57	4	4	0	8	4	6	1	11	19
102	2002	2	14.07.02	75	8	6	0	14	3	4	1	8	22
102	2003	1	19.05.03	19	5	3	0	8	3	2	0	5	13
102	2003	2	08.07.03	69	9	7	0	16	10	4	3	17	33
102	2004	1	12.05.04	12	3	2	0	5	5	1	0	6	11
102	2004	2	16.07.04	77	3	2	0	5	3	4	1	8	13
102	2005	1	18.05.05	18	13	3	0	16	2	9	1	12	28
102	2005	2	01.08.05	93	5	5	0	10	3	14	4	21	31
102	2006	1	14-31.05.06	22	13	3	0	16	4	14	0	18	34
102	2006	2	09.07.06	70	9	2	0	11	12	15	2	29	40
102	2007	1	27.05.07	27	7	4	0	11	13	11	1	25	36
102	2007	2	02.-03.07.07	63	7	6	0	13	5	18	1	24	37
108	2002	1	13.06.02	44	11	3	0	14	0	0	1	1	15
108	2002	2	02.07.02	63	3	2	0	5	5	2	5	12	17
108	2003	1	27.05.03	27	8	11	0	19	6	12	4	22	41
108	2003	2	30.06.03	61	15	10	0	25	3	7	1	11	36
108	2004	1	27.05.04	27	5	10	0	15	1	16	5	22	37
108	2004	2	17.07.04	78	7	13	0	20	6	10	2	18	38
108	2005	1	13.05.05	13	5	7	1	13	1	6	2	9	22
108	2005	2	01.08.05	93	4	7	0	11	3	12	5	20	31
108	2006	1	14.-21.05.06	20	11	5	0	16	3	11	5	19	35
108	2006	2	11.07.06	72	8	6	0	14	6	10	4	20	34
108	2007	1	27.05.07	27	16	9	0	25	5	6	1	12	37
108	2007	2	05.-07.07.07	66	12	9	0	21	6	12	3	21	42
111	2002	1	03.06.02	34	2	3	2	7	0	1	12	13	20
111	2002	2	21.06.02	52	2	3	0	5	1	5	9	15	20
111	2003	1	10.05.03	10	2	3	1	6	1	12	16	29	35

111	2003	2	06.07.03	67	1	4	0	5	2	16	17	35	40
111	2004	1	02.06.04	33	6	10	1	17	4	5	4	13	30
111	2004	2	20.-22.07.04	82	4	6	1	11	0	11	8	19	30
111	2005	1	22.05.05	22	8	30	1	39	4	9	4	17	56
111	2005	2	04.08.05	96	3	7	1	11	1	16	2	19	30
111	2006	1	13.-31.05.06	22	13	16	0	29	3	9	1	13	42
111	2006	2	16.-17.07.06	77	9	14	0	23	3	21	3	27	50
111	2007	1	30.05-03.06.07	31	0	7	1	8	2	13	8	23	31
111	2007	2	08.07.07	69	3	9	1	13	2	14	7	23	36
126	2002	1	15.06.02	46	4	3	2	9	3	4	13	20	29
126	2002	2	29.06.02	60	1	5	0	6	0	6	8	14	20
126	2003	1	11.-12.05.03	11	8	12	0	20	3	19	49	71	91
126	2003	2	18.06.03	49	5	17	1	23	4	6	16	26	49
126	2004	1	13.05.04	13	0	7	0	7	3	7	18	28	35
126	2004	2	13.07.04	74	4	15	1	20	1	15	14	30	50
126	2005	1	11.05.05	11	6	7	0	13	0	7	9	16	29
126	2005	2	04.08.05	96	0	4	0	4	6	8	13	27	31
126	2006	1	13.-31.05.06	26	12	9	0	21	6	9	1	16	37
126	2006	2	16.-17.07.06	77	6	14	0	20	7	17	4	28	48
126	2007	1	03.06.07	34	8	12	0	20	3	11	7	21	41
126	2007	2	03.07.07	64	4	7	0	11	7	13	13	33	44
134	2002	1	29.05.02	29	4	2	0	6	9	3	1	13	19
134	2002	2	15.07.02	76	1	5	0	6	3	4	6	13	19
134	2003	1	16.05.03	16	4	4	0	8	5	5	2	12	20
134	2003	2	25.06.03	56	5	12	0	17	8	16	4	28	45
134	2004	1	30.05.04	30	4	9	0	13	3	16	2	21	34
134	2004	2	12.07.04	73	1	14	0	15	0	8	0	8	23
134	2005	1	18.-20.05.05	20	2	7	0	9	3	18	1	22	31
134	2005	2	07.08.05	99	1	11	0	12	2	17	0	19	31
134	2006	1	07.-08.06.06	38	7	5	0	12	7	13	0	20	32
134	2006	2	22.07.06	83	5	12	0	17	6	16	0	22	39
134	2007	1	25.-26.05.07	26	2	11	0	13	7	10	0	17	30
134	2007	2	07.-16.07.07	75	8	9	0	17	5	11	1	17	34
138	2002	1	30.05.02	30	0	2	2	4	0	2	15	17	21
138	2002	2	13.06.02	44	0	2	0	2	0	3	9	12	14
138	2003	1	29.05.03	29	2	4	1	7	0	6	21	27	34
138	2003	2	10.07.03	71	0	4	1	5	1	2	38	41	46
138	2004	1	28.05.04	28	1	4	2	7	1	2	29	32	39
138	2004	2	24.07.04	85	0	2	2	4	0	3	26	29	33
138	2005	1	17.05.05	17	1	5	0	6	1	13	9	23	29
138	2005	2	03.08.05	95	0	3	1	4	0	12	17	29	33
138	2006	1	09.06.06	40	2	10	0	12	0	11	13	24	36
138	2006	2	21.-23.07.06	83	1	4	0	5	1	16	8	25	30
138	2007	1	24.05.07	24	1	7	1	9	0	20	6	26	35
138	2007	2	12.-15.07.07	74	1	12	1	14	2	26	6	34	48
Total					868	1085	86	2039	548	1354	1110	3012	5051
