# Supplementary material for: Coexistence of diploid and triploid hybrid water frogs: population differences persist in the apparent absence of differential survival
Source: BMC Ecol. 2010 May 27;10:14. doi: 10.1186/1472-6785-10-14 (PMC2902419; doi:10.1186/1472-6785-10-14)
Supplement: Additional file 2 — AICc weights for different models of p in the 12 ponds keeping Φ constant. Table of AICc weights. [file 1472-6785-10-14-S2.PDF]

Additional file 2. AICc weights for different models of p in the 12 ponds keeping Φ constant.

Model	001	011	014	032	032A	089 [1)]	102	108	111 [1)]	126	134	138 [1)]	Mean
Φ(.) p(genotype*sex*time)	0.0000	0.0000	0.0000	0.0000	0.0000	0.0000	0.0000	0.0000	0.0000	0.0000	0.0000	0.0000	0.0000
Φ(.) p(genotype*sex*season)	0.0039	0.0220	0.0081	0.0000	0.0000	0.0005	0.1028	0.0009	0.0264	0.0032	0.0403	0.0000	0.0173
Φ(.) p(genotype*sex)	0.3055	**0.3588**	0.0685	0.0001	0.0002	0.0002	0.0108	0.0729	0.0289	0.0152	0.0320	0.0027	0.0746
Φ(.) p(genotype*time)	0.0000	0.0000	0.0000	0.0000	0.0000	0.0000	0.0000	0.0000	0.0000	0.0000	0.0000	0.0000	0.0000
Φ(.) p(genotype*season)	0.0024	0.0278	0.0984	0.0000	0.0001	0.0033	0.2302	0.0066	0.0374	0.0427	0.0181	0.0008	0.0390
Φ(.) p(sex*time)	0.0000	0.0241	0.0002	0.0005	0.0024	0.0471	0.0000	0.0000	0.0061	0.0011	0.0047	0.0002	0.0072
Φ(.) p(sex*season)	0.0877	0.1318	0.0331	0.0005	0.0002	0.0203	**0.2887**	0.0377	**0.7283**	0.2962	0.0054	0.0011	0.1359
Φ(.) p(genotype)	0.0366	0.0488	0.2446	0.0001	0.0008	0.0004	0.0050	0.1432	0.0495	0.0440	0.0097	0.0041	0.0489
Φ(.) p(sex)	**0.5082**	0.3188	0.0494	0.0004	0.0008	0.0016	0.0751	0.1423	0.0346	0.1169	0.0060	0.0023	0.1047
Φ(.) p(time)	0.0007	0.0043	**0.3520**	**0.9966**	**0.9947**	**0.8663**	0.0022	0.0650	0.0003	0.0031	**0.8618**	**0.9736**	0.4267
Φ(.) p(season)	0.0166	0.0380	0.0846	0.0013	0.0002	0.0584	0.2615	0.1410	0.0256	**0.4068**	0.0169	0.0088	0.0883
Φ(.) p(.)	0.0384	0.0258	0.0611	0.0006	0.0006	0.0020	0.0238	**0.3904**	0.0630	0.0709	0.0051	0.0065	0.0573

Grey marks AICc weights for models where the ΔAICc (data not shown) was <2.

Bold marks the best model per pond.

[1)] data includes LRR males in these ponds.
