# Supplementary material for: Coexistence of diploid and triploid hybrid water frogs: population differences persist in the apparent absence of differential survival
Source: BMC Ecol. 2010 May 27;10:14. doi: 10.1186/1472-6785-10-14 (PMC2902419; doi:10.1186/1472-6785-10-14)
Supplement: Additional file 3 — AICc weights for different models of Φ in the 12 ponds using previously identified best models for p. Table of AICc weights. [file 1472-6785-10-14-S3.PDF]

Additional file 3. AICc weights for different models of Φ in the 12 ponds using the previously identified best models for p (2nd row).

	001	001	011	011	014	014	032	032A	089 1)	102	102	108	111 1)	126	134	138 1)
Model p ->	sex	geno*sex	geno*sex	sex	time	geno	time	time	time	sex*season	season	.	sex*season	season	time	time
Φ(geno*sex*time)	0.1031	**0.8970**	**1.0000**	1E-05	0	0	-	**1**	**1**	-	-	**0.9604**	**0.9998**	**1**	0	**0.9798**
Φ(geno*sex*season)	0	0	0	0	0.0006	0.0003	0.0010	0	0	0.0004	0.0005	0.0003	0	0	0.0172	0.0002
Φ(geno*sex)	0	0	0	0	0.0059	0.0022	0.0367	0	0	0.01173	0.0200	0.0157	0.00002	0	0.1382	0.0025
Φ(geno*time)	0	0	-	1E-05	0	0	0	7E-05	0	0.00015	9E-05	0.0010	0.00004	-	0.0926	0.0006
Φ(geno*season)	0	0	0	0	0.0195	0.0079	0.0115	0	0	0.00424	0.0059	0.0008	0	0	0.0419	0.0005
Φ(sex*time)	0	0	0	0	0	0.0001	0	0	0	0	0	0.0032	0	0	0	0
Φ(sex*season)	0	0	0	0	0.0237	0.0176	0.0890	0	0	0.01525	0.0223	0.0008	0.00003	0	0.0201	0.0007
Φ(geno)	0	0	0	0	0.0285	0.0098	0.1253	0	0	0.05014	0.0441	0.0134	0.00001	0	0.0472	0.0084
Φ(sex)	0	0	0	0	0.0355	0.0243	0.1455	0	0	0.08439	0.1451	0.0013	0.00002	0	0.1493	0.0040
Φ(time)	0	0	0	0	0.0003	0.4907	0.0017	0	0	0.00043	0.0006	4E-05	0	0	0.0001	0
Φ(season)	0	0	0	0	**0.1097**	0.0948	0.2478	0	0	0.09069	0.0793	0.0009	0.00002	0	0.1283	0.0011
Φ(.)	0	0	0	0	0.0759	0.0527	**0.3415**	0	0	**0.23927**	0.1854	0.0021	0.00006	0	**0.3651**	0.0023

Grey marks AICc weights for models where the ΔAICc (data not shown) was <2.

Bold marks the best model per pond.

"-" means that the analysis did not converge.

1) data includes LRR males in these ponds.

geno is an abbreviation for genotype
